# Supplementary material for: Discovery and application of insertion-deletion (INDEL) polymorphisms for QTL mapping of early life-history traits in Atlantic salmon
Source: BMC Genomics. 2010 Mar 8;11:156. doi: 10.1186/1471-2164-11-156 (PMC2838853; doi:10.1186/1471-2164-11-156)
Supplement: Additional file 2 — Information on developed 76 locus single-run INDEL panel in Atlantic salmon. Information on fluorescence labeling, primer concentrations, PCR pooling and links to alignments, INDEL motifs and GENESCAN (Burge and Karlin 1997) predictions of genes/exons are available in html format. [file 1471-2164-11-156-S2.ZIP › Additionalfile2/table17363.html]

Contig Alignment

```
Base	A B C D E F   
1	. G . . . .    
2	. G . . . .    
3	. G . . . .    
4	. A . . . .    
5	. T . . . .    
6	. G . . . .    
7	. A . . . .    
8	. G . . . .    
9	. T . . . .    
10	. G . . . .    
11	. T . . . .    
12	. C . . . .    
13	. T . . . .    
14	. C . . . .    
15	. T . . . .    
16	. T . . . .    
17	. T . . . .    
18	. T . . . .    
19	. T . . . .    
20	. T . . . .    
21	. C . . . .    
22	. C . . . .    
23	. A . . . .    
24	. G . . . .    
25	. T . . . .    
26	. T . . . .    
27	. T . . . .    
28	. T . . . .    
29	. T . . . .    
30	. G . . . .    
31	. T . . . .    
32	. T . . . .    
33	. T . . . .    
34	. T . . . .    
35	. A . . . .    
36	. C . . . .    
37	. T . . . .    
38	. C . . . .    
39	. T . . . .    
40	. C . C . .    
41	. A . A . .    
42	. G . G . .    
43	. A . A . .    
44	. G . G . .    
45	. A . A . .    
46	. T . T . .    
47	. G . G . .    
48	. G . G . .    
49	. T . T . .    
50	. T . T . .    
51	. T . T . .    
52	. T . T . .    
53	. A . A . .    
54	. A . A . .    
55	. T . T . .    
56	. G . G . .    
57	. T . T . .    
58	. C . C . .    
59	. A . A . .    
60	. A . A . .    
61	. A . A . .    
62	. T . T . .    
63	. T . T . .    
64	. T . T . .    
65	. G . G . .    
66	. A . A . .    
67	. A . A . .    
68	. T . T . .    
69	. G . G . .    
70	. T . T . .    
71	. A . A . .    
72	. G . G . .    
73	. G . G . .    
74	. G . G . .    
75	. T . T . .    
76	. G . G . .    
77	. T . T . .    
78	. T . T . .    
79	. T . T . .    
80	. T . T . .    
81	. T . T . .    
82	. G . G . .    
83	. T . T . .    
84	. G . G . .    
85	. T . T . .    
86	. T . T . .    
87	. G . G . .    
88	. T . T . .    
89	. G . G . .    
90	. A . A . .    
91	. G . G . .    
92	. T . T . .    
93	. C . C . .    
94	. T . T . .    
95	. G . G . .    
96	. T . T . .    
97	. T . T . .    
98	. T . T . .    
99	. A . A . .    
100	. T . T . .    
101	. A . A . .    
102	. A . A . .    
103	. C . C . .    
104	. T . T . .    
105	. G . G . .    
106	. T . T . .    
107	. A . A . .    
108	. A . A . .    
109	. A . A . .    
110	. T . T . .    
111	. A . A . .    
112	. C . C . .    
113	. A . A . .    
114	. T . T . .    
115	. T . T . .    
116	. G . G . .    
117	. A . A . .    
118	. A . A . .    
119	. T . T . .    
120	. G . G . .    
121	. T . T . .    
122	. A . A . .    
123	. T . T . .    
124	. G . G . .    
125	. T . T . .    
126	. T . T . .    
127	. G . G . .    
128	. C . C . .    
129	. T . T . .    
130	. T . T . .    
131	. G . G . .    
132	. A . A . .    
133	. T . T . .    
134	. T . T . .    
135	. G . G . .    
136	. T . T . .    
137	. A . A . .    
138	. G . G . .    
139	. A . A . .    
140	. C . C . .    
141	. T . T . .    
142	. T . T . .    
143	. T . T . .    
144	. T . T . .    
145	. A . A . .    
146	. C . C . .    
147	. A . A . .    
148	. T . T . .    
149	. T . T . .    
150	. C . C . .    
151	. T . T . .    
152	. T . T . .    
153	. A . A . .    
154	. A . A . .    
155	. C . C . .    
156	. A . A . .    
157	. C . C . .    
158	. A . A . .    
159	. A . A . .    
160	. A . A . .    
161	. C . C . .    
162	. C . C . .    
163	. T . T . .    
164	. A . A . .    
165	. A . A . .    
166	. G . G . .    
167	. C . C . .    
168	. T . T . .    
169	. C . C . .    
170	. T . T . .    
171	. C . C . .    
172	. A . A . .    
173	. C . C . .    
174	. C . C . .    
175	. T . T . .    
176	. G G G . .    
177	. A A A . .    
178	. G G G . .    
179	. C C C . .    
180	. A A A . .    
181	. A A A . .    
182	. C C C . .    
183	. A A A . .    
184	. C C C . .    
185	. T T T . .    
186	. G G G . .    
187	. C C C . .    
188	. C C C . .    
189	. T T T . .    
190	. G G G . .    
191	. C C C . .    
192	. A A A . .    
193	. A A A . .    
194	. C C C . .    
195	. C C C . .    
196	. A A A . .    
197	. C C C . .    
198	. A A A . .    
199	. T T T . .    
200	. G G G . .    
201	. T T T . .    
202	. T T T . .    
203	. C C C . .    
204	. A A A . .    
205	. A A A . .    
206	. T T T . .    
207	. T T T . .    
208	C C C C C .    
209	A A A A A .    
210	G G G G G .    
211	C C C C C .    
212	A A A A A .    
213	C C C C C .    
214	T T T T T .    
215	T T T T T T    
216	T T T T T T    
217	T T T T T T    
218	T T T T T T    
219	C C C C C C    
220	A A A A A A    
221	C C C C C C    
222	T T T T T T    
223	T T T T T T    
224	T T T T T T    
225	T T T T T T    
226	T T T T T T    
227	T T T T T T    
228	C C C C C C    
229	A A A A A A    
230	G G G G G G    
231	T T T T T T    
232	T T T T T T    
233	T T T T T T    
234	A A A A A A    
235	A A A A A A    
236	A A A A A A    
237	T T T T T T    
238	G G G G G G    
239	T T T T T T    
240	T T T T T T    
241	T T T T T T    
242	C C C C C C    
243	A A A A A A    
244	G G G G G G    
245	G G G G G G    
246	C C C C C C    
247	C C C C C C    
248	A A A A A A    
249	T T T T T T    
250	G G G G G G    
251	A A A A A A    
252	C C C C C C    
253	C C C C C C    
254	C C C C C C    
255	A A A A A A    
256	G G G G G G    
257	G G G G G G    
258	A A A A A A    
259	G G G G G G    
260	G G G G G G    
261	C C C C C C    
262	C C C C C C    
263	C C C C C C    
264	T T T T T T    
265	A A A A A A    
266	C C C C C C    
267	T T T T T T    
268	G G G G G G    
269	T T T T T T    
270	A A A A A A    
271	G G G G G G    
272	C C C C C C    
273	C C C C C C    
274	C C C C C C    
275	A A A A A A    
276	G G G G G G    
277	G G G G G G    
278	C C C C C C    
279	C C C C C C    
280	C C C C C C    
281	A A A A A A    
282	C C C C C C    
283	C C C C C C    
284	G G G G G G    
285	T T T T T T    
286	C C C C C C    
287	G G G G G G    
288	C C C C C C    
289	T T T T T T    
290	A A A A A A    
291	T T T T T T    
292	T T T T T T    
293	G G G G G G    
294	G G G G G G    
295	A A A A A A    
296	A A A A A A    
297	C C C C C C    
298	C C C C C C    
299	A A A A A A    
300	G G G G G G    
301	T T T T T T    
302	G G G G G G    
303	C C C C C C    
304	T T T T T T    
305	A A A A A A    
306	G G G G G G    
307	G G G G G G    
308	A A A A A A    
309	C C C C C C    
310	A A A A A A    
311	T T T T T T    
312	G G G G G G    
313	G G G G G G    
314	G G G G G G    
315	T T T T T T    
316	A A A A A A    
317	A A A A A A    
318	T T T T T T    
319	G G G G G G    
320	T T T T T T    
321	T T T T T T    
322	G G G G G G    
323	A A A A A A    
324	G G G G G G    
325	G G G G G G    
326	A A A A A A    
327	C C C C C C    
328	T T T T T T    
329	T T T T T T    
330	T T T T T T    
331	A A A A A A    
332	C C C C C C    
333	T T T T T T    
334	A A A A A A    
335	A A A A A A    
336	A A A A A A    
337	A A A A A A    
338	T T T T T T    
339	A A A A A A    
340	T T T T T T    
341	T T T T T T    
342	T T T T T T    
343	C C C C C C    
344	T T T T T T    
345	T T T T T T    
346	G G G G G G    
347	A A A A A A    
348	T T T T T T    
349	T T T T T T    
350	C C C C C C    
351	T T T T T T    
352	C C C C C C    
353	C C C C C C    
354	C C C C C C    
355	A A A A A A    
356	C C C C C C    
357	C C C C C C    
358	G G G G G G    
359	A A A A A A    
360	C C C C C C    
361	A A A A A A    
362	G G G G G G    
363	A A A A A A    
364	C C C C C C    
365	A A A A A A    
366	G G G G G G    
367	A A A A A A    
368	G G G G G G    
369	G G G G G G    
370	G G G G G G    
371	T T T T T T    
372	G G G G G G    
373	G G G G G G    
374	A A A A A A    
375	A A A A A A    
376	T T T T T T    
377	G G G G G G    
378	A A A A A A    
379	T T T T T T    
380	A A A A A A    
381	T T T T T T    
382	A A A A A A    
383	G G G G G G    
384	A A A A A A    
385	T T T T T T    
386	G G G G G G    
387	T T T T T T    
388	A A A A A A    
389	T T T T T T    
390	C C C C C C    
391	T T T T T T    
392	G G G G G G    
393	T T T T T T    
394	A A A A A A    
395	G G G G G G    
396	A A A A A A    
397	T T T T T T    
398	A A A A A A    
399	C C C C C C    
400	A A A A A A    
401	A A A A A A    
402	A G A G A A  2	 transition  
403	C C C C C C    
404	A A A A A A    
405	G G G G G G    
406	A A A A A A    
407	C C C C C C    
408	A A A A A A    
409	A A A A A A    
410	G G G G G G    
411	T T T T T T    
412	A A A A A A    
413	T T T T T T    
414	G G G G G G    
415	C C C C C C    
416	C C C C C C    
417	G G G G G G    
418	T T T T T T    
419	G G G G G G    
420	A A A A A A    
421	G G G G G G    
422	A A A A A A    
423	G G G G G G    
424	A A A A A A    
425	G G G G G G    
426	A A A A A A    
427	G G G G G G    
428	G G G G G G    
429	G G G G G G    
430	G G G G G G    
431	T T T T T T    
432	C C C C C C    
433	T T T T T T    
434	G G G G G G    
435	G G G G G G    
436	A A A A A A    
437	C C C C C C    
438	A A A A A A    
439	G G G G G G    
440	G G G G G G    
441	G G G G G G    
442	T T T T T T    
443	T T T T T T    
444	G G G G G G    
445	T T T T T T    
446	C C C C C C    
447	A A A A A A    
448	G G G G G G    
449	T T T T T T    
450	T T T T T T    
451	T T T T T T    
452	A A A A A A    
453	G G G G G G    
454	C C C C C C    
455	C C C C C C    
456	G G G G G G    
457	C C C C C C    
458	T T T T T T    
459	T T T T T T    
460	C C C C C C    
461	A A A A A A    
462	A A A A A A    
463	T T T T T T    
464	A A A A A A    
465	G G G G G G    
466	A A A A A A    
467	G G G G G G    
468	G G G G G G    
469	A A A A A A    
470	G G G G G G    
471	A A A A A A    
472	A A A A A A    
473	T T T T T T    
474	T T T T T T    
475	A A A A A A    
476	T T T T T T    
477	C C C C C C    
478	T T T T T T    
479	G G G G G G    
480	C C C C C C    
481	A A A A A A    
482	G G G G G G    
483	A A A A A A    
484	T T T T T T    
485	G G G G G G    
486	T T T T T T    
487	T T T T T T    
488	T T T T T T    
489	T T T T T T    
490	T T T T T T    
491	G G G G G G    
492	G G G G G G    
493	G G G G G G    
494	G G G G G G    
495	A A A A A A    
496	A A A A A A    
497	G G G G G G    
498	A A A A A A    
499	A A A A A A    
500	T T T T T T    
501	T T T T T T    
502	G G G G G G    
503	T T T T T T    
504	T T T T T T    
505	T T T T T T    
506	A A A A A A    
507	C C C C C C    
508	A A A A A A    
509	C C C C C C    
510	A A A A A A    
511	G G G G G G    
512	C C C C C C    
513	C C C C C C    
514	C C C C C C    
515	A A A A A A    
516	T T T T T T    
517	A A A A A A    
518	C C C C C C    
519	A A A A A A    
520	A A A A A A    
521	T T T T T T    
522	C C C C C C    
523	A A A A A A    
524	T T T T T T    
525	G G G G G G    
526	A A A A A A    
527	C C C C C C    
528	T T T T T T    
529	C C C C C C    
530	C C C C C C    
531	A A A A A A    
532	G G G G G G    
533	A A A A A A    
534	T T T T T T    
535	A A A A A A    
536	G G G G G G    
537	T T T T T T    
538	G G G G G G    
539	T T T T T T    
540	T T T T T T    
541	T T T T T T    
542	G G G G G G    
543	T T T T T T    
544	G G G G G G    
545	T T T T T T    
546	G G G G G G    
547	G G G G G G    
548	G G G G G G    
549	C C C C C C    
550	T T T T T T    
551	G G G G G G    
552	T T T T T T    
553	G G G G G G    
554	G G G G G G    
555	A A A A A A    
556	G G G G G G    
557	G G G G G G    
558	C C C C C C    
559	G G G G G G    
560	A A A A A A    
561	C C C C C C    
562	G G G G G G    
563	G G G G G G    
564	T T T T T T    
565	T T T T T T    
566	A A A A A A    
567	C C C C C C    
568	C C C C C C    
569	A A A A A A    
570	T T T T T T    
571	G G G G G G    
572	G G G G G G    
573	A A A A A A    
574	C C C C C C    
575	A A A A A A    
576	C C C C C C    
577	T T T T T T    
578	C C C C C C    
579	A A A A A A    
580	C C C C C C    
581	A A A A A A    
582	C C C C C C    
583	T T T T T T    
584	A A A A A A    
585	A A A A A A    
586	A A A A A A    
587	T T T T T T    
588	C C C C C C    
589	A A A A A A    
590	T T T T T T    
591	T T T T T T    
592	C C C C C C    
593	A A A A A A    
594	T T T T T T    
595	C C C C C C    
596	A A A A A A    
597	A A A A A A    
598	T T T T T T    
599	A A A A A A    
600	C T T T C T  2	 transition  
601	G G G G G G    
602	A A A A A A    
603	A A A A A A    
604	T T T T T T    
605	C C C C C C    
606	A A A A A A    
607	G G G G G G    
608	A A A A A A    
609	C C C C C C    
610	T T T T T T    
611	C C C C C C    
612	A A A A A A    
613	T T T T T T    
614	G G G G G G    
615	C C C C C C    
616	A A A A A A    
617	C C C C C C    
618	G G G G G G    
619	A A A A A A    
620	G G G G G G    
621	T T T T T T    
622	C C C C C C    
623	C C C C C C    
624	A A A A A A    
625	A A A A A A    
626	C C C C C C    
627	T T T T T T    
628	G G G G G G    
629	C C C C C C    
630	T T T T T T    
631	C C C C C C    
632	T T T T T T    
633	C C C C C C    
634	A A A A A A    
635	T T T T T T    
636	G G G G G G    
637	T T T T T T    
638	T T T T T T    
639	C C C C C C    
640	A A A A A A    
641	T T T T T T    
642	G G G G G G    
643	T T T T T T    
644	A A A A A A    
645	G G G G G G    
646	A A A A A A    
647	A A A A A A    
648	T T T T T T    
649	T T T T T T    
650	A A A A A A    
651	G G G G G G    
652	C C C C C C    
653	C C C C C C    
654	T T T T T T    
655	G G G G G G    
656	G G G G G G    
657	C T T T T T    
658	. T T T T T    
659	. G G G G G    
660	. C C C C C    
661	. C C C C C    
662	. G G G G G    
663	. T T T T T    
664	. C C C C C    
665	. T T T T T    
666	. A A A A A    
667	. T T T T T    
668	. T T T T T    
669	. T T T T T    
670	. T T T T T    
671	. C C C C C    
672	. A A A A A    
673	. G G G G G    
674	. C C C C C    
675	. T T T T T    
676	. A A A A A    
677	. T T T T T    
678	. T T T T T    
679	. T T T T T    
680	. C C C C C    
681	. A A A A A    
682	. T T T T T    
683	. T T T T T    
684	. C C C C C    
685	. C C C C C    
686	. A A A A A    
687	. C C C C C    
688	. T T T T T    
689	. T T T T T    
690	. C C C C C    
691	. T T T T T    
692	. T T T T T    
693	. G G G G G    
694	. T T T T T    
695	. A A A A A    
696	. C C C C C    
697	. T T T T T    
698	. C C C C C    
699	. T T T T T    
700	. G G G G G    
701	. T T T T T    
702	. G - G - G  2	 indel  
703	. T - T - T  2	 indel  
704	. A A A A A    
705	. A A A A A    
706	. T T T T T    
707	. T T T T T    
708	. T T T T T    
709	. G G G G G    
710	. C C C C C    
711	. C C C C C    
712	. A A A A A    
713	. A A A A A    
714	. A A A A A    
715	. G G G G G    
716	. A A A A A    
717	. G G G G G    
718	. G G G G G    
719	. G G G G G    
720	. G G G G G    
721	. C C C C C    
722	. A A A A A    
723	. T T T T T    
724	. A A A A A    
725	. C C C C C    
726	. T T T T T    
727	. C C C C C    
728	. T T T T T    
729	. T T T T T    
730	. T T T T T    
731	. T T T T T    
732	. G G G G G    
733	. G G G G G    
734	. T T T T T    
735	. A A A A A    
736	. . A A A A    
737	. . A A A A    
738	. . T T T T    
739	. . G G G G    
740	. . A A A A    
741	. . C C C C    
742	. . A A A A    
743	. . T T T T    
744	. . G G G G    
745	. . T T T T    
746	. . G G G G    
747	. . T T T T    
748	. . G G G G    
749	. . G G G G    
750	. . C T C C    
751	. . A A A A    
752	. . A A A A    
753	. . G G G G    
754	. . T T T T    
755	. . A A A A    
756	. . G G G G    
757	. . T T T T    
758	. . G G G G    
759	. . G G G G    
760	. . A A A A    
761	. . A A A A    
762	. . T T T T    
763	. . G G G G    
764	. . C C C C    
765	. . T T T T    
766	. . A A A A    
767	. . G G G G    
768	. . C C C C    
769	. . T T T T    
770	. . A A A A    
771	. . A A A A    
772	. . A A A A    
773	. . A A A A    
774	. . C C C C    
775	. . G G G G    
776	. . A A A A    
777	. . C C C C    
778	. . T T T T    
779	. . A A A A    
780	. . G G G G    
781	. . T T T T    
782	. . A A A A    
783	. . C C C C    
784	. . T T T T    
785	. . C C C C    
786	. . T T T T    
787	. . G G G G    
788	. . A A A A    
789	. . C C C C    
790	. . T T T T    
791	. . A A A A    
792	. . A A A A    
793	. . T T T T    
794	. . G G G G    
795	. . T T T T    
796	. . A A A A    
797	. . G G G G    
798	. . A A A A    
799	. . A A A A    
800	. . C C C C    
801	. . G G G G    
802	. . G G G G    
803	. . A A A A    
804	. . A A A A    
805	. . T T T T    
806	. . C C C C    
807	. . T T T T    
808	. . G G G G    
809	. . T T T T    
810	. . C C C C    
811	. . T T T T    
812	. . G G G G    
813	. . T T T T    
814	. . A A A A    
815	. . A A A A    
816	. . G G G G    
817	. . T T T T    
818	. . A A A A    
819	. . A A A A    
820	. . G G G G    
821	. . T T T T    
822	. . G G G G    
823	. . T T T T    
824	. . G G G G    
825	. . T T T T    
826	. . G G G G    
827	. . T T T T    
828	. . G G G G    
829	. . G T T T    
830	. . . G G G    
831	. . . T T T    
832	. . . A A A    
833	. . . C C C    
834	. . . A A A    
835	. . . A A A    
836	. . . T T T    
837	. . . A A A    
838	. . . A A A    
839	. . . A A A    
840	. . . G G G    
841	. . . A A A    
842	. . . C C C    
843	. . . A A A    
844	. . . C C C    
845	. . . T T T    
846	. . . G G G    
847	. . . T T T    
848	. . . C C C    
849	. . . C C C    
850	. . . A A A    
851	. . . A A A    
852	. . . A A A    
853	. . . T T T    
854	. . . G G G
```
